# Helicobacter hepaticus, a recently recognized bacterial pathogen, associated with chronic hepatitis and hepatocellular neoplasia in laboratory mice.

**DOI:** 10.3201/eid0104.950404

**Published:** 1995

**Authors:** J M Rice

**Affiliations:** Laboratory of Comparative Carcinogenesis, National Cancer Institute, Frederick, Maryland, USA.


### *Helicobacter hepaticus*, a Recently Recognized Bacterial Pathogen, Associated with Chronic Hepatitis and Hepatocellular Neoplasia in Laboratory Mice

Gastric carcinoma, one of the most prevalent human cancers worldwide, is among the neoplasms for which epidemiologic evidence of environmental causes is strongest. The exact nature of these environmental causes was obscure until mounting evidence recently linked chronic infection of the gastric antrum mucosa by *Helicobacter pylori* (a microaerobic, gram-negative, spiral bacterium) with elevated cancer risk (1). It is now recognized that gastric B-cell lymphoma of mucosa-associated lymphoid tissue is also closely linked to gastric *H. pylori* infection, and eradication of the infection with antibiotics can result in regression of the lymphoma (2,3). This startling finding has stimulated intense interest in the genus *Helicobacter* and related organisms; as a result, additional species of *Helicobacter* are now frequently isolated and characterized from many non-human hosts. Until 1994, however, only *H. pylori* was known to be associated with tumor development, in humans or in any other animal species.

In 1992, at the National Cancer Institute's Frederick Cancer Research and Development Center (FCRDC) in Frederick, Maryland, a high prevalence of liver disease was observed among certain strains of mice; these mice were untreated controls in long-term chemical carcinogenesis experiments. Affected strains, notably A/JCr, had been bred at FCRDC under pathogen-free conditions and were free of known serologically detectable murine viruses and parasites; moreover, they had no histologically demonstrable hepatic abnormalities, except for a very low incidence (1% to 2%) of hepatocellular tumors in mice 15 months of age or older. Over a very short period, the prevalence of a histologically distinctive form of hepatitis increased to virtually 100% in male mice at 1 year of age (Table 1). The earliest demonstrable lesions were small, undistinctive foci of hepatic necrosis seen in young mice aged 2 to 6 months. In older mice, aged 6 to 10 months, there was a highly distinctive pericholangitis, consisting of abundant mononuclear cell infiltrates around bile ducts within portal triads. The biliary epithelium within affected ducts was focally swollen, and the luminal surfaces of damaged epithelial cells were poorly defined in hematoxylin and eosin-stained sections (4,5). In livers with extensive lesions, bile ductular (oval cell) hyperplasia was also prominent. Moreover, mice with hepatitis usually had hepatocellular tumors, often multiple, that included both adenomas and carcinomas (4).

Hepatocellular tumors in mice are one of the most common endpoints in bioassays for chemical carcinogens. They were not, at that time, known to be associated with infectious agents. Accordingly, initial efforts to identify the cause of the hepatitis/hepatocellular tumor syndrome were directed toward possible sources of chemical exposure. The possibility of accidental exposure to experimental substances within the research animal facilities was ruled out when liver disease was identified in mice that had never left the breeding areas which are located in separate buildings. Extensive chemical analyses of food, bedding, water, and other possible sources of toxic substances had negative results.

Detailed pathologic examination by light microscopy of tissue sections from diseased livers was continued, and many special stains were used. One such stain, Steiner's silver impregnation procedure for spirochetes (6), revealed in hepatic tissue uniform bodies that were consistent in size and shape with bacteria. Homogenates of fresh liver tissue from diseased mice proved effective in transmitting hepatitis to A/J mice purchased from commercial sources outside FCRDC, when given by intraperitoneal injection (5). In addition, from these homogenates, a motile, spiral bacterium could be cultivated on blood agar plates incubated at 37$^{o}$ C under anaerobic or microaerobic conditions.

This organism was subsequently characterized by ultrastructural morphologic examination, biochemical characteristics, and 16S rRNA gene sequence. Determined to be a new species related to *H. pylori*, it was given the name *H. hepaticus* (7). The bacterium is motile and gram negative, 0.2 to 0.3 µm in diameter, 1.5 to 5.0 µm long, and curved to spiral in shape, with one to several spirals; it has bipolar sheathed flagella (one at each end) but

Table 1. Increasing prevalence of hepatitis and hepatocellular neoplasia in control male A/JCr mice at the National Cancer Institute's Frederick Cancer Research and Development Center, 1989-1992[a]

| Date killed | Number of mice | Age, in weeks | Mice with hepatitis (%) | Mice with liver tumors (%) |
|---|---|---|---|---|
| Jan-Mar 1989 | 48 | 47±3 | 0 | 0 |
| May-Jul 1989 | 47 | 70±60 | 0 | 1 (2) |
| Jan-Apr 1992 | 6 | 36±4 | 2 (33) | 0 |
| Jul 1992 | 16 | 54 | 16 (100) | 1 (6) |
| Aug-Oct 1992 | 6 | 64±3 | 5 (83) | 3 (50) |
| Dec 1992 | 12 | 77 | 12 (100) | 11 (92) |

[a]Adapted from ref. 4.

lacks the periplasmic fibers that envelope the bacterial cells in other mouse *Helicobacter* species. *H. hepaticus* has strong urease activity, is oxidase and catalase positive, produces $H_2S$, reduces nitrate to nitrite, and grows microaerobically at $37^o$ C but not at $25^o$ C or $42^o$ C. It is resistant to cephalothin and nalidixic acid but sensitive to metronidazole. Photographs illustrating its morphologic structure by light (4,5) and electron (4,5,7) microscopy have been published. The species-defining characteristic of the organism, the nucleotide sequence of its 16S rRNA gene, has been used to develop a diagnostic assay based on polymerase chain reaction (8).

Systematic examination of rodents of all species and strains produced at FCRDC, especially retired breeders, showed that the characteristic hepatitis and associated bacteria were present in mice of several strains (A/JCr, DBA/2NCr, C3H/HeNCr) and that within these strains, the male mice were more severely affected than the female. Mice with severe combined immunodeficiencies were especially vulnerable. The precise location of organisms demonstrable by Steiner stain within infected liver parenchyma was shown by transmission electron microscopy to be invariably extracellular and characteristically within bile canaliculi (4,5). No liver disease was seen in some strains (e.g., C57BL/6NCr) or in F1 hybrids between sensitive and resistant strains (e.g., B6C3F1). Rodent species other than mice (e.g., rats, Syrian hamsters, and guinea pigs) were not affected.

In infected mice with severe combined immunodeficiency, cecal inflammation was histologically demonstrable (5), and organisms were isolated from the mucosa of the large intestine (7), which may mean that the usual ecologic niche occupied by *H. hepaticus* is that of a commensal colonizer of the intestinal tract (8). Since mice are coprophagic, it appears highly likely that natural transmission of the organisms is the oral-fecal route. Why and how *H. hepaticus* invades the liver in mice of certain strains remain to be determined. Hepatitis is also characteristic of certain other enteric pathogenic bacteria, such as *Campylobacter jejuni* (9) that, unlike *H. hepaticus,* have not been associated with liver tumor development. The tissue damage that accompanies persistent infection by *H. hepaticus, H. pylori,* and certain other *Helicobacter* species may be due, at least in part, to a soluble, trypsin-sensitive cytotoxin of high molecular weight produced by these organisms (10). There is no precedent for any direct role of such a toxin in carcinogenesis. On the other hand, chronic infections by viruses, bacteria, or certain parasites are recognized risk factors for human cancers at various sites. The hypothesis that chemically reactive, potentially genotoxic, substances of low molecular weight (including nitric oxide and active oxygen species) generated by inflammatory cells at the site of chronic infection may initiate or enhance carcinogenesis has been examined (11). The hypothesis is under active investigation in the context of *H. hepaticus*-associated liver disease.

*H. hepaticus* is susceptible to a number of antibiotics; treatment of susceptible, naturally infected 8- to 10-week-old strain A/JCr mice with single or combined antimicrobial agents has been evaluated for efficacy in eradicating established infections (12). Amoxicillin, metronidazole, and tetracycline administered singly failed to eradicate bacteria from the gastrointestinal tract, but either amoxicillin or tetracycline, in combination with metronidazole and bismuth, was effective in eradicating *H. hepaticus* from the liver, cecum, and colon when given by oral gavage for a period of 2 weeks (12). The effect of antibiotic therapy on the carcinogenic process, or in older animals, remains to be established.

The importance of *H. hepaticus* to humans is not yet completely known. The organism clearly has the potential to confound bioassays for chemical carcinogens, but this potential has no direct effect on humans. Even though most *Helicobacter* species identified to date are characteristically associated with (and named after) specific mammalian host species in which they generally inhabit the gastrointestinal tract (with or without causing gastritis or other chronic inflammatory disease), the potential host range for some species is quite broad. *H. pylori,* originally isolated from humans, has recently been isolated also from the domestic cat; this raises the possibility that *Helicobacter pylori* may be a zoonotic pathogen that can be transmitted from companion animals to humans (13). Exploring the possibility of zoonotic transmission of *H. pylori*, *H. hepaticus,* or any other *Helicobacter* species would require isolation of the organism in question by culture methods. Serologic methods have not yet been refined to the level of species specificity. Humans infected with *H. pylori* mount a serum antibody response to the bacteria that is readily detected by enzyme-linked immunosorbent assays and is considered evidence of ongoing disease (1); mice infected with *H. hepaticus* similarly produce serum antibodies to that species that have been demonstrated by Western blotting (5). Antisera to *H. pylori* can be used to visualize *H. hepaticus* in mouse liver tissue sections stained by the avidin-biotin complex immunohistochemical procedure (5). The cross-reactivity between these two species precludes use of available serologic methods to establish whether *H. hepaticus* has infected humans.

Regardless of whether *H. hepaticus* is itself capable of infecting humans, it serves to demonstrate that liver tissue can be persistently infected by at least one member of the genus *Helicobacter*, and that liver cancer can be a long-term consequence of such infection. This discovery raises questions about the existence of a comparable relationship between liver cancer in humans and unrecognized bacterial infections. Reviews are under way of tissue blocks from pathology archives in search of organisms demonstrable by the Steiner stain in liver sections from human populations at high risk for liver cancer.

**Jerry M. Rice**
Laboratory of Comparative Carcinogenesis, National Cancer Institute, Frederick, Maryland, USA

### References

1. Parsonnet J, Friedman GD, Vandersteen DP, Chang Y, Vogelman JH, Orentreich N, et al. *Helicobacter pylori* infection and the risk of gastric carcinoma. N Engl J Med 1991;325:1127-31.
2. Wotherspoon AC, Doglioni C, Diss TC, Pan L, Moschini A, de Boni M, et al. Regression of primary low-grade B-cell gastric lymphoma of mucosa-associated lymphoid tissue type after eradication of *Helicobacter pylori*. Lancet 1993;342:575-7.
3. Bayerdörffer E, Neubauer A, Rudolph B, Thiede C, Lehn N, Eidt S, et al. Regression of primary gastric lymphoma of mucosa-associated lymphoid tissue type after cure of *Helicobacter pylori* infection. Lancet 1995;345:1591-4.
4. Ward JM, Fox JG, Anver MR, Haines DC, George CV, Collins MJ Jr, et al. Chronic active hepatitis and associated liver tumors in mice caused by a persistent bacterial infection with a novel *Helicobacter* species. J Natl Cancer Inst 1994;86:1222-7.
5. Ward JM, Anver MR, Haines DC, Benveniste RE. Chronic active hepatitis in mice caused by *Helicobacter hepaticus*. Am J Pathol 1994;145:959-68.
6. Garvey W, Fathi A, Bigelow F. Modified Steiner for the demonstration of spirochetes. J Histotechnology 1985;8:15-7.
7. Fox JG, Dewhirst FE, Tully JG, Paster BJ, Yan L, Taylor NS, et al. *Helicobacter hepaticus* sp. nov., a microaerophilic bacterium isolated from livers and intestinal mucosal scrapings from mice. J Clin Microbiol 1994;32:1238-45.
8. Battles JK, Williamson JC, Pike KM, Gorelick PL, Ward JM, Gonda MA. Diagnostic assay for *Helicobacter hepaticus* based on nucleotide sequence of its 16S rRNA gene. 1995;33:1344-7.
9. Kita E, Oku D, Hamuro A, Nishikawa F, Emoto M, Yagyu Y, et al. Hepatotoxic activity of *Campylobacter jejuni*. J Med Microbiol 1990;33:171-82.
10. Taylor NS, Fox JG, Yan L. In-vitro hepatotoxic factor in *Helicobacter hepaticus, H. pylori* and other *Helicobacter* species. J Med Microbiol 1995;42:48-52.
11. Ohshima H, Bartsch H. Chronic infections and inflammatory processes as cancer risk factors: possible role of nitric oxide in carcinogenesis. Mutat Res 1994;305:253-64.
12. Foltz CJ, Fox JG, Yan L, Shames B. Evaluation of antibiotic therapies for eradication of *Helicobacter hepaticus*. Antimicrob Agents Chemother 1995;39:1292-4.
13. Handt LK, Fox JG, Dewhirst FE, Fraser GJ, Paster BJ, Yan LL, et al. *Helicobacter pylori* isolated from the domestic cat: public health implications. Infect Immun 1994;62:2367-74.
